# Body composition in male hypogonadism: practical considerations to the use of dual-energy x-ray absorptiometry

**DOI:** 10.1007/s11154-026-10054-5

**Published:** 2026-05-25

**Authors:** Andrea Delbarba, Myriam Amer, Walter Vena, Biagio Cangiano, Emanuele Ferrante, Alessandro Pizzocaro, Alessandro Chilà, Marilina Romeo, Valeria Lanzi, Caterina Buoso, Giorgio Tiecco, Matteo Riva, Alfredo Berruti, Gherardo Mazziotti, Alberto Ferlin, Marco Bonomi

**Affiliations:** 1 Department of Clinical and Experimental Sciences, SSD Endocrinologia, University of Brescia, ASST Spedali Civili, Brescia, Italy; 2 Endocrinology, Diabetology and Medical Andrology Unit, IRCCS Humanitas Research Hospital, Milan, Italy; 3 Department of Biomedical Sciences, Humanitas University, Milan, Italy; 4 Unit of Endocrinology & Diabetes, Humanitas Gavazzeni Institute, Bergamo, Italy; 5 Department of Medical Biotechnology and Translational Medicine, University of Milan, Milan, Italy; 6 Department of Endocrine and Metabolic Diseases, IRCCS Istituto Auxologico Italiano, Milan, Italy; 7 Endocrinology Unit, Fondazione IRCCS Ca’ Granda Ospedale Maggiore Policlinico, Milan, Italy; 8 Department of Clinical Sciences and Community Health, University of Milan, Milan, Italy; 9 Department of Clinical and Experimental Sciences, Unit of Infectious and Tropical Diseases, University of Brescia and ASST Spedali Civili di Brescia, Brescia, Italy; 10 Department of Medical and Surgical Specialties, Radiological Sciences and Public Health, University of Brescia, ASST Spedali Civili of Brescia, Brescia, Italy; 11 Medical Oncology Unit, Department of Medical and Surgical Specialties, Radiological Sciences and Public Health, University of Brescia at ASST Spedali Civili, Brescia, Italy; 12 Department of Medicine, University of Padova, Padua, Italy; 13 Unit of Andrology and Reproductive Medicine, Department of Systems Medicine, University Hospital of Padova, Padua, Italy; 14 IRCCS Istituto Auxologico Italiano, Via Mosè Bianchi 90, Milano, 20149 Italy

**Keywords:** Body composition, Male hypogonadism. Kallmann’s syndrome, Klinefelter’s syndrome, Transgender AMAB, PLWH, Androgen Deprivation Therapy

## Abstract

Male hypogonadism is associated with significant alterations in body composition, including reduced lean body mass (LBM), increased fat body mass (FBM), particularly visceral adiposity, and impaired muscle function, contributing to frailty and cardiometabolic risk. These changes reflect the disruption of a complex endocrine crosstalk among bone, muscle, and adipose tissue, mediated by cytokines such as osteokines, myokines, and adipokines. This dysregulation promotes the development of osteosarcopenic obesity, a condition characterized by the coexistence of low bone mass, sarcopenia, and excess adiposity. Testosterone (T) plays a central role in maintaining body composition by stimulating muscle protein synthesis, inhibiting adipogenesis, and preserving bone health. Its deficiency, irrespective of etiology, leads to rapid impairment of anabolic pathways, resulting in decreased lean mass and increased fat accumulation. Evidence from clinical and experimental models demonstrates that these alterations are partially reversible with T replacement therapy (TRT), although variability exists depending on the underlying cause of hypogonadism. Dual-energy X-ray absorptiometry (DXA) represents the gold standard for assessing bone mineral density (BMD) and a key tool for evaluating body composition through a three-compartment model. It allows precise quantification of fat and lean mass, as well as their regional distribution, with minimal radiation exposure. In this review, we provide a comprehensive and clinically oriented overview of body composition alterations in male hypogonadism, focusing on underlying pathophysiological mechanisms and the practical application of DXA across different clinical scenarios. We discuss evidence from conditions such as Klinefelter syndrome, Kallmann syndrome, androgen deprivation therapy, HIV infection, and transgender care, aiming to offer a pragmatic framework for integrating body composition assessment into routine practice and improving patient management.

**Supplementary Information:**

The online version contains supplementary material available at 10.1007/s11154-026-10054-5.

## Introduction

### Bone, muscle and adipose tissue: a complex crosstalk

In recent years, driven by the global rise in aging populations and the obesity pandemic, the assessment of body composition (BC) has emerged as a subject of growing scientific investigation and clinical application [1]. Indeed, historically the main idea was that BC was just the partition of different tissue contents, and that muscle-bone relationship was only settled for the mechano-transduction’s function. However, not only the two largest tissues of the musculoskeletal system are physically connected enhancing each other their bio-mechanical role, but they are also deeply involved in the human BC [2, 3], together with the adipose tissue. This complex interplay between organs, tissues and cells is the groundwork through which organisms undergo growth and development [4]. Indeed, each of the three tissues can produce, distribute and deliver nutrients and other substances. Apart from the already known hormonal link, mainly involving sex steroids and growth hormone-insulin-like growth factor 1 (GH-IGF1), these tissues are inter-connected via other substances [5]. Specifically, bone contributes through the secretion of osteokines, derived from bone cells [6], myocytes lead to the secretion of myokines [7] and, finally, adipose tissue secrets adipokines from adipocytes [3]. Such molecules belong to the family of cytokines, which act in autocrine, paracrine and endocrine manner, and are regulated by several factors, such as aging and exercise [8]. Bone cells secret a broad range of osteokines which influence not only bone remodeling, but they also exert beneficial effects on glucose metabolism and muscle performance, influencing liver and skeletal muscle homeostasis [6, 9]. While human studies on osteocalcin still lack robust evidence, mouse models have shown its positive metabolic effects, with lipolytic, anti-adipogenic and anti-diabetogenic properties [5]. Sclerostin, on the other hand, acts as a Wnt signaling antagonist in pre-osteoblasts, suppressing their differentiation, thus inhibiting bone formation [5]. Beyond its skeletal effects, emerging evidence suggests that sclerostin may also contribute to muscle impairment, as supported by cross-sectional data showing an association with reduced muscle mass in individuals with sarcopenia [10]. This supports a role for sclerostin as a potential mediator linking bone metabolism and muscle loss. Skeletal muscle, likewise, secretes different myokines, such as irisin, myostatin and brain-derived neurotrophic factor (BDNF), in response to exercise, modulating both adipose tissue metabolism and bone growth generating a tightly regulated signaling axis linking bone, muscle and adipose tissue [11]. Myokines released during exercise, such as irisin, contribute to the browning of white adipose tissue and promote energy expenditure [3], whereas myonectin facilitates fatty acid uptake by adipose tissue and liver [12]. In contrast, myostatin acts as a muscle-specific inhibitory myokine that decreases with exercise and exerts a strong negative effect on muscle growth [12]. Furthermore, it induces adipocytes differentiation and contributes to insulin resistance [13]. Hence, it can be seen as a mediator of the pathological link between sarcopenia and obesity [3, 12]. Still playing a role in this cytokines-rich orchestra, adipokines secreted by adipose tissue, namely leptin and adiponectin, together with their ratio, are considered valuable markers of insulin resistance and cardiometabolic risk in childhood obesity [14]. Leptin has important functions in appetite regulation and energy metabolism; in particular, it reduces appetite and promotes energy expenditure through the activation of pro-opiomelanocortin (POMC)-expressing neurons and the inhibition of the of the hypothalamus agouti-related protein (AgRP) and neuropeptide Y (NPY) [12]. Conversely, adiponectin – inversely associated with FBM – is known for its insulin-sensitizing and anti-inflammatory effects [5]; adiponectin is also expressed by skeletal muscle [15], where it is potentially induced by endurance training [5, 16]. Bearing this in mind, it emerges how these three systems are biochemically interdependent, generating a tightly signaling axis that extends far beyond their local function. These interactions vary across the lifespan, and their disruption contributes to a wide range of diseases, including chronic inflammation, obesity, osteoporosis, sarcopenia and the combination of the three [2].

### Osteosarcopenic obesity syndrome

Osteosarcopenic obesity (OSO) is a syndrome in which there is the coexistence of reduced bone mass (osteopenia/osteoporosis), reduced muscle mass (sarcopenia), and excess adipose tissue (overweight/obesity). Another feature of this syndrome is the fat redistribution toward the visceral compartment and adipogenesis within bone and muscle tissues [17]. Adipose-tissue alterations can arise even when body mass index (BMI) values are below the diagnostic cut off for overweight, so the term osteosarcopenic adiposity (OSA) has been proposed as more appropriate for this syndrome [18]. The assessments that allow us to define the diagnostic criteria of OSO are of two types: structural, through the use of body-composition measurements obtained with DXA or bioelectrical impedance analysis (BIA); functional, via physical-performance measures (handgrip strength, single-leg stance, gait speed, and the chair sit-to-stand test) [19]. Cutoffs for osteopenia/osteoporosis are well defined, while those for sarcopenia and adiposity are more heterogeneous. Osteopenia and osteoporosis are defined by lumbar spine and total hip T-scores of < − 1.0 and < − 2.5, respectively. Sarcopenia can be assessed via Skeletal Muscle Index, SMI (≤ 7.26 kg/m² men, ≤ 5.45 kg/m² women) or Appendicular Lean Mass, ALM (< 20 th percentile). Overweight/obesity is commonly defined by BMI (25–30 kg/m^2^ and > 30 kg/m²), though alternative measures, such as total body fat (> 25% men, > 32% women), Fat Mass Index (> 9 kg/m² men, > 13 kg/m² women), or visceral-to-subcutaneous fat ratio > 1, may be more accurate [20, 21]. The absence of standardized criteria complicates recognition of OSO as a distinct entity, and this makes it more difficult to adequately evaluate its clinical impact compared to each of the components considered separately [22]. Prevalence estimates vary widely depending on the population under study and diagnostic criteria applied, ranging from 6% to over 90% [23]. In the meta-analysis by Ying et al. [24], OSO prevalence was approximately 8%, while acknowledging substantial heterogeneity across studies. From this and other work, women appear to have a higher incidence than men [25, 26], and physical inactivity is associated with increased OSO risk [26]. Multiple factors are implicated in OSO pathogenesis. One is low-grade chronic inflammation which contributes to osteoporosis, sarcopenia, and adiposity through pro-inflammatory cytokines (IL-6, IL-1, and TNF-α) [18]. Adipokines also play an important role: adiponectin promotes bone formation and stimulates myogenesis, with serum levels correlating negatively with FBM; leptin, under physiological conditions, plays a beneficial role on bones and muscles, improving osteoblastic activity and myogenesis, whereas leptin resistance appears to contribute substantially to OSO. There are also other mediators involved in the muscle-bone-adipose cross-talk, such as chemerin and Early B-Cell Factor-1 (Ebf1), which are still under study [21]. These factors act on mesenchymal stem cells, the precursors of the three cellular lineages underlying the condition (adipocytes, osteoblasts, and myocytes), promoting adipocytic differentiation within muscle and bone as well [18, 21]. Another pathogenic mechanism is linked to the lack of regulation of various endocrine axes, involving glucocorticoids, GH and IGF-1, and sex hormones. The beneficial effects of androgens on LBM, FBM, and bone health are well recognized [24, 27]. Therapeutic strategies for OSO focus on resistance exercise, dietary modifications (adequate daily protein and calcium intake), and vitamin D supplementation in deficient individuals. Pharmacological therapy is aimed at the individual components of OSO, employing agents approved for obesity management and osteoporosis treatment [23, 27–29].

### Impact of hypogonadism on body composition

Male hypogonadism has a profound impact on body composition, primarily through reduction in lean mass and increase in FBM [30]. T plays a central role in promoting lean mass accrual, in fact, it regulates muscle protein synthesis, mitochondrial function, satellite cell activation, and inhibits catabolic pathways; consequently, androgen deficiency rapidly disrupts the balance between anabolic and catabolic signalling independently from aetiology of hypogonadism [31]. As already described, T normally stimulates myogenesis and muscle hypertrophy by enhancing protein synthesis and suppressing protein degradation, while promoting satellite cell differentiation [32]. T decline impairs these pathways, resulting in loss of lean body mass and progressive muscle weakness [32]. Observational studies in boys with hypogonadism have reported an unfavourable lean-to-fat ratio compared with age-matched healthy controls, with lower lean mass and higher FBM in affected subjects. These findings support a role for T in the regulation of body composition during development; however, given the heterogeneity of age and pubertal status in the available cohorts, the precise timing of these alterations across developmental stages remains uncertain [33]. Clinical models of induced hypogonadism provide clear evidence of this effect: men undergoing androgen deprivation therapy for prostate cancer (PC) experience a 3.8% reduction in lean body mass and an 11% increase in FBM within 12 months [32]. Muscle cross-sectional area decreases by 15–22% in key muscle groups, demonstrating how rapidly anabolic processes are compromised in the absence of adequate T signalling [34]. Evidence in older men demonstrates that T deficiency correlates strongly with sarcopenia, reduced muscle strength, and decreased physical performance [33]. These findings highlight T as a critical regulator of muscle mass and functional capacity throughout life [32]. Beyond muscle loss, hypogonadism promotes adiposity, with a preferential increase in visceral fat [34]. The accumulation of visceral adipose tissue is not only a mechanical consequence of reduced energy expenditure and lower muscle mass but also involves endocrine dysregulation [34]. Declining T levels enhance adipocyte differentiation and favour lipid storage, while visceral fat expansion fuels chronic inflammation through increased secretion of IL-6 and TNF-α, cytokines that have direct catabolic effects on skeletal muscle, thus perpetuating sarcopenia [32]. This vicious circle of androgen deficiency and visceral adiposity is also well documented in men with functional hypogonadism related to obesity, where adipose tissue expansion contributes to suppression of the hypothalamic-pituitary-gonadal axis and further T decline [35]. Observational studies consistently show an association between circulating T, LBM, and FBM. In the New Mexico Aging Process Study and other large cohorts, lower free T correlates with lower appendicular muscle mass and reduced muscle strength [32]. Similarly, population studies in China and the United States demonstrate a positive association between T levels and appendicular lean mass, and an inverse relationship with FBM [32]. Together, these findings support a dose-response relationship between T concentration and body composition parameters. Intervention studies further clarify the role of T in modulating body composition, showing that T therapy can reduce visceral and subcutaneous fat mass, waist circumference as well as increase lean mass [36–45]. Data from a recent clinical trial in which hypogonadal men were treated with T cypionate show that T replacement improves fat-free mass across the entire cohort, regardless of baseline levels; however, men with baseline T < 264 ng/dL (*reference range 350–1000*) experienced a significantly greater increase in total and appendicular lean mass [46]. These findings suggest that individuals with more profound androgen deficiency exhibit greater anabolic responsiveness once eugonadism is re-established. Conversely, improvements in FBM and metabolic parameters may be more dependent on baseline metabolic health than on the degree of hypogonadism [46]. Hypogonadism secondary to obesity is a unique phenotype of androgen deficiency where body composition alterations and hormonal changes reinforce each another. Men with severe obesity and low T display higher body weight, BMI, and body fat percentage than eugonadal obese controls [35]. Notably, weight loss, whether spontaneous or following bariatric surgery, often leads to normalization of T levels and parallel improvements in body composition, emphasizing that adiposity itself plays a pivotal role in driving endocrine dysfunction [35, 47]. This variability highlights the heterogeneous phenotypes of hypogonadism and the need to distinguish primary, congenital, and functional etiologies when interpreting body composition markers. Differences in BMD between individuals with hypergonadotropic and hypogonadotropic hypogonadism have been described in the literature. Notably, the only available comparative study, although including patients with different forms of hypogonadism, reported lower BMD values in those with hypergonadotropic hypogonadism. These observations may point to a potential role of extragonadal actions of follicle-stimulating hormone (FSH), beyond its classical reproductive functions [48]. Elevated FSH levels could contribute to adverse skeletal outcomes through direct effects on bone metabolism, as suggested by the presence of functional FSH receptors on osteoclasts [49]. In addition, high circulating FSH levels have been proposed as a possible mediator of unfavourable changes in body composition, including increased FBM and reduced LBM, which may further exacerbate skeletal fragility [50]. Alternative and possibly complementary mechanisms should also be considered. Impaired Leydig cell function, a hallmark of hypergonadotropic hypogonadism, may negatively influence bone health and body composition through reduced insulin-like factor 3 (INSL3) secretion and disturbances in vitamin D metabolism [51]. Together, these mechanisms may contribute to the observed differences in BMD and highlight the complex interplay between gonadal dysfunction and the musculoskeletal system [52]. In conclusion, hypogonadism promotes a typical body composition profile characterized by reduced LBM, increased FBM, especially visceral, and decreased muscle strength. These changes contribute to functional decline, frailty, and increased metabolic risk. DXA is central for quantifying body composition, detecting altered patterns, and monitoring responses to TRT or weight-loss interventions.

### DXA for BMD and body composition assessment

Dual energy X-ray absorptiometry (DXA) is a radiological technique based on the use of X-rays at two different energy levels. As the X-ray beams pass through the anatomical regions of interest, tissue attenuation is measured; through the application of subtraction algorithms, this approach allows differentiation between soft tissues and bone tissue [53]. DXA is one of the most accurate and precise methods for quantifying bone mineral mass and density in vivo. The DXA system creates a planar (two-dimensional) image resulting from the combination of low- and high-energy attenuations. From these planar images, bone mineral content (BMC) can be estimated, expressed in grams. By defining a region of interest (ROI), BMC can be normalized to the scanned area to calculate BMD, expressed in g/cm² [54]. The selection of anatomical ROIs is standardized and typically includes:

- lumbar spine (L1–L4) in anteroposterior projection: one ROI per vertebra;
- pelvis including both femurs: four ROIs on one side, preferably the non-dominant side, including the femoral neck, greater trochanter, intertrochanteric region, and Ward’s triangle; for diagnostic purposes, the lowest value is considered;
- proximal radius of the non-dominant limb (rarely used): indicated when spine and hip measurements are unreliable due to artifacts (e.g., severe osteoarthritis, metallic implants, or vertebroplasty, which may falsely elevate BMD), in conditions such as hyperparathyroidism (due to predominant cortical bone involvement), or in cases of severe obesity (BMI > 35).

ROI placement must strictly adhere to the manufacturer’s guidelines [55]. BMD represents the primary quantitative parameter derived from DXA and can be compared with reference population data to calculate either the Z-score or the T-score. The Z-score is referenced to an age-, sex-, and ethnicity-matched population, whereas the T-score is referenced to the mean BMD of a healthy young adult population [54, 55]. Whole-body DXA extends the analyses described above to the entire body, allowing a comprehensive assessment of body composition (BC). DXA-based body composition analysis relies on three-compartment model that distinguishes bone, FBM, and LBM, and assumes that X-ray transmission through tissues follows a mono-exponential absorption law, which is proportional to the molecular weight of the molecules composing the tissue. The use of two X-ray beams at different energy levels, based on this three-compartment model, allows the calculation of tissue composition for each pixel of the image matrix, which is represented by a specific grayscale value. This is achieved by solving a system of two equations with two variables, enabling the distinction between bone tissue and soft tissue. In regions where bone tissue is absent, this method allows further differentiation between adipose tissue and fat-free tissue (i.e., muscle tissue); therefore, soft tissue differentiation must be performed in regions without bone tissue [55]. DXA uses ionizing radiation. According to the American College of Radiology, the effective dose for a DXA examination is approximately 0.001 mSv, comparable to about 3 h of natural background radiation. This dose is roughly 100 times lower than that of a standard chest X-ray (approximately 0.1 mSv, equivalent to about 10 days of background radiation) [54]. The dosimetric assessment of a total body DXA examination shows wide variability depending on the patient’s body composition, the type of equipment used, and the technical acquisition settings. The literature reports an effective dose range between 0.001 and 0.075 mSv, which is nonetheless lower than that of a standard chest radiograph [56].

#### BMD interpretation

For diagnostic classification, the BMD value is converted into a T-score and/or Z-score, representing the patient’s BMD in standard deviations relative to a reference population, as previously specified, and reported with a precision of one decimal place. The T-score is used in postmenopausal women and in men aged 50 years or older. For the diagnosis of osteoporosis, only the T-score is considered; when multiple T-scores are available, the lowest value is used for diagnostic classification [57]. According to the World Health Organization (WHO) criteria, a T-score ≤ − 2.5 is consistent with a diagnosis of osteoporosis, whereas T-score values ≥ − 1.0 at these regions of interest indicate normal BMD. A T-score between − 2.5 and − 1.0 is defined as osteopenia, also referred to as low bone mass or low bone density [57]. The Z-score is primarily used in pediatric patients, who have not yet achieved peak bone mass, as well as in men younger than 50 years and in premenopausal women. In these younger populations, a Z-score ≤ − 2.0 is defined by the International Society for Clinical Densitometry (ISCD) as “BMD below the expected range for age”, and the use of the terms osteopenia or osteoporosis is discouraged for BMD-based classification [58].

### Body composition parameters interpretation

Using the three-compartment model, body composition parameters are derived, particularly FBM and LBM. From skeletal muscle mass (SMM, kg) and FBM (kg), various derived parameters can be calculated, including body size–adjusted indices (e.g., lean mass index) and indices of fat distribution (e.g., android-to-gynoid ratio, trunk-to-leg fat percentage ratio, and trunk-to-limb FBM ratio). The various body composition parameters obtained from whole-body DXA are reported and described in the Supplementary Table 1. DXA scanning can also be used to assess the distribution of visceral adipose tissue (VAT), although this estimation is based on a two-dimensional projection of the body. While it is not considered the gold-standard technique, DXA allows the estimation of VAT, showing good correlation with measurements obtained using gold-standard imaging techniques such as abdominal magnetic resonance imaging (MRI) and computed tomography (CT) [59]. While the potential of DXA for body composition analysis has long been acknowledged, this application has only recently been systematically investigated; consequently, there remains limited consensus regarding the use of standardized cut-off values for body composition parameters [60]. However, in recent years, some guidelines have proposed reference values to be used in clinical practice (summarized in Table 1). Most guidelines recommend adjusting lean mass for body size; however, there is ongoing debate regarding the most appropriate adjustment method and whether a single approach is suitable for all populations. It is important to emphasize that reduced values of these indices indicate a condition of low muscle mass. Nevertheless, the diagnosis of sarcopenia requires the simultaneous presence of reduced muscle strength, as determined through specific tests (e.g. hand grip test, chair stand test) [61, 62]. In 2019, the European Working Group on Sarcopenia in Older People (EWGSOP) updated its clinical algorithm for sarcopenia and provided clear cut-off values for measurements used to identify and characterize sarcopenia [61]. EWGSOP2 adopted a cut-off of 20 kg for ALM, a reference value primarily proposed by the Foundation for the National Institutes of Health (FNIH) Sarcopenia Project, — an American research initiative that analyzed data from nine sources involving community-dwelling older adults [63]. Additionally, EWGSOP2 set a cut-off of 7.0 kg/m² for ALM adjusted for height squared (ALM/H^**2**^), based on reference ranges proposed in a large Australian population-based study [64]. The most recent guidelines were issued in 2022 in a joint consensus statement by the European Society for Clinical Nutrition and Metabolism (ESPEN) and the European Association for the Study of Obesity (EASO), focusing on sarcopenic obesity [62]. The panel recommended adjusting lean mass for body weight, specifically suggesting the use of SMM adjusted for weight (SMM/W) and ALM adjusted for body weight (ALM/W). The proposed cut-off values for males are 37% for SMM/W, based on reference ranges reported by Janssen et al. [65], and 25.7% for ALM/W, as indicated by Batsis et al. [66]. The EASO and ESPEN guidelines on sarcopenic obesity also proposed reference values for FBM (FBM%) stratified by age groups and ethnicity. The cut-off values for male population are extrapolated from the study by Gallagher et al. [67] and are detailed in Table 1. For other parameters (e.g. VAT, fat mass index, android-to-gynoid ratio), although reference values have been proposed in various publications, there are currently no guideline-validated cut-off points.

**Table 1:** Validated cut-off values for sarcopenic obesity diagnosis in men

Index	Cut-off points for men	Guideline/Consensus reference
ALM Appendicular Skeletal Muscle Mass	< 20 Kg	EWGSOP European consensus [60]
ALM/H^2^ Appendicular Lean Mass/Height^2^	< 7 Kg/m^2^	EWGSOP European consensus [60]
SMM/W Skeletal Lean Mass/Weight	CLASS I of Sarcopenia: 31.5–37% CLASS II of Sarcopenia: <31.5%	ESPEN and EASO Consensus Statement [61]
ALM/W Appendicular Lean Mass/Weight	< 25.7%	ESPEN and EASO Consensus Statement [61]
FM Fat Mass	*20-39y*: > 26% (Caucasians); >28% (Asians); >26% (African-Americans) *40–59 y*: > 29% (Caucasians); >29% (Asians); >27% (African-Americans); *60-79y*: > 31% (Caucasians); >29% (Asians); >29% (African-Americans);	ESPEN and EASO Consensus Statement [61]

### Additional analyses

**Fig. 1:**
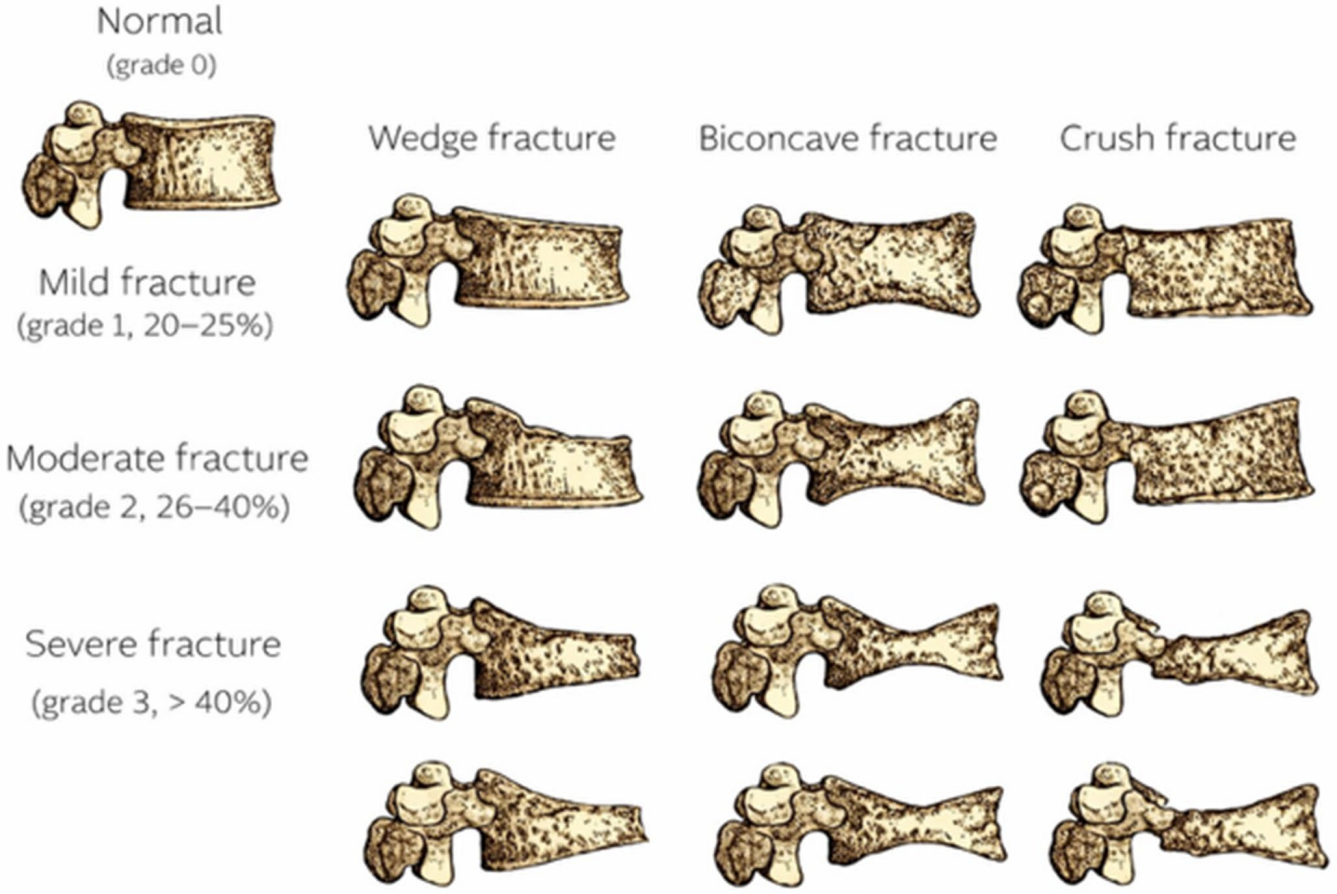
Genant classification of vertebral fractures. Semi-quantitative method for grading vertebral deformities based on vertebral height reduction and morphological changes. Grades include: grade 0 (normal), grade 1 (mild, 20–25% reduction), grade 2 (moderate, 26–40% reduction), and grade 3 (severe, > 40% reduction). Adapted from [67]

In addition to the traditional DXA scan, a lateral spine projection for vertebral fracture assessment (VFA) can be added. VFA can be performed using single- or dual-energy techniques and allows evaluation of vertebrae from T5 to L4. DXA-based VFA is associated with a lower radiation dose and reduced parallax error compared with standard X-ray; however, it has a longer acquisition time and lower spatial resolution. Therefore, it can be used only for vertebral fracture diagnosis based on Genant’s classification, and no additional assessments can be performed on these images (e.g., evaluation of anatomical anomalies or BMD calculation) [55]. According to Genant’s criteria, a grade 1 deformity is defined as a 20–25% reduction in anterior, middle, and/or posterior vertebral height; a grade 2 deformity is defined as a 26–40% reduction in any vertebral height; and a grade 3 deformity is defined as a reduction greater than 40% in any vertebral height, Fig. 1 [68].

At the vertebral level, dedicated software allows the evaluation of additional parameters, such as the trabecular bone score (TBS) and the bone strain index (BSI), which contribute to the assessment of fracture risk and bone quality. TBS is a recently defined parameter that can be derived from a lumbar spine DXA image. Through an analysis of bone texture (obtained by evaluating grayscale matrix), TBS provides an indirect assessment of bone microarchitecture, which correlates with mechanical properties. TBS is an independent predictor of fracture risk; however, it should not be used as a standalone tool for fracture risk assessment. Degraded TBS was categorized according to data from a meta-analysis [69]: values below 1.23 define degraded TBS, values between 1.23 and 1.31 define partially degraded TBS, and values above 1.31 define normal TBS. The software can also calculate a BMD T-score adjustment based on TBS. The addition of TBS assessment to FRAX and/or BMD enhances fracture risk prediction in both primary and secondary osteoporosis, providing valuable information for treatment decision-making and monitoring [70]. BSI is a recently defined parameter that can be obtained from DXA images using specific software [71]. It provides information on vertebral bone quality; however, unlike BMD, which considers the amount of bone mineral, and TBS, which considers the distribution of mineral mass, BSI assesses the vertebra’s ability to withstand mechanical loads. The vertebra is considered as an element subjected to different types of loads (compressive, rotational, and flexural), and through grayscale texture analysis, BSI provides data on density distribution, bone geometry, and load resistance in localized areas. The BSI value therefore represents the local load stress experienced by the bone; higher values indicate an increased risk of fracture (standardized cut-off values are not yet validated) [72].

## Clinical evidence of the impact of body composition on bone health

### Klinefelter syndrome

Klinefelter syndrome (KS) is the most common sex chromosome aneuploidy in males, typically caused by one or more supernumerary X chromosomes [73]. Beyond reproductive features, KS is associated with multiple comorbidities, including altered body composition, metabolic disorders, and increased skeletal fragility, with a higher risk of osteopenia, osteoporosis and fractures [31]. Several studies have assessed BMD with DXA, reporting a prevalence of osteoporosis ranging from 6% to 15% and of osteopenia from 25% to 48% in KS adults [31, 74]. In this context, while hypogonadism only partially explains reduced BMD and fracture risk [75], recent studies have demonstrated that increased adiposity, particularly visceral fat, and reduced lean mass are strongly associated with impaired bone quality in this population [76, 77]. As reported in other forms of secondary osteoporosis, KS patients may experience fragility fractures despite having normal BMD, highlighting the limited reliability of DXA in accurately assessing skeletal health and fracture risk in this setting [71]. Interestingly, novel imaging-derived parameters, such as BSI, which reflects bone deformation under mechanical load, have emerged as promising tools for fracture risk assessment [78]. Indeed, in a recent study by Pigni et al. [76], lumbar BSI (L-BSI) was strongly correlated with markers of adiposity, including BMI, fat mass index (FMI), fat-to-lean mass ratio, and visceral adipose tissue. Higher FBM was associated with impaired TBS and greater L-BSI, indicating reduced bone quality and microarchitecture and mechanical strength. Interestingly, these associations were independent of serum T, estradiol (E2), vitamin D, or duration of TRT, suggesting that unfavourable body composition exerts a direct detrimental effect on skeletal integrity. No association was found between L-BSI, TBS and body composition parameters and vertebral fractures, probably due to the limited sample number. Furthermore, patients with a later diagnosis of KS had higher adiposity and worse L-BSI values, underlining the importance of early detection and management. In KS patients, bone microarchitecture has also been investigated using high-resolution peripheral quantitative computed tomography (HR-pQCT), which demonstrated lower trabecular density and a reduction in cortical bone area [79]. Recently, a multicenter cross-sectional study investigated the relationship between body composition, TBS and vertebral fractures (VFs) in 71 KS adult men [80]. Despite a relatively young median age (41 years), nearly one in five patients presented with VFs (19%), which occurred independently of BMD. Alterations in body composition were prominent: increased FBM, particularly central adiposity, and reduced lean mass were common findings. Impaired TBS, reflecting altered trabecular microarchitecture, was observed in 26% of subjects and correlated strongly with higher body mass index, waist circumference, FMI, and fat-to-lean mass ratio. Importantly, VFs were significantly associated with an increased truncal/leg fat ratio, suggesting that fat distribution, rather than absolute weight or serum T levels, plays a central role in skeletal fragility. Neither T replacement therapy nor vitamin D status significantly influenced skeletal outcomes. Taken together, these findings support the concept of a dual adverse effect of excess body fat in KS, whereby fat-derived proinflammatory cytokines and altered bone–fat crosstalk promote microarchitectural deterioration, while mechanical strain properties are compromised. This evidence underscores the importance of considering body composition as a major determinant of skeletal health in KS and suggests that targeted interventions to reduce adiposity may represent a novel therapeutic avenue to improve bone quality and prevent fractures in this high-risk population. In a recent article comparing patients with KS and Kallmann syndrome, rare form of genetic central hypogonadism (see also below) the analysis of the entire cohort demonstrated an independent association between Appendicular Lean Mass Index (ALMI) and both lumbar and total hip BMD, confirming the role of lean mass in bone health. Moreover, among all the parameters analyzed that may modulate muscle mass, only FSH showed a statistically significant inverse association, independent of T levels. Although no difference in terms of BMD was found between the two populations studied, ALMI was found to be statistically lower in the Klinefelter group, suggesting greater fragility in this population [50].

### Congenital hypogonadotropic hypogonadism (CHH)

CHH is a rare form of central hypogonadism characterized by a GnRH secretion or action defect that could be associated or not with a reduced or absent sense of smell respectively in the Kallmann syndrome or normosmic-CHH. Its prevalence is approximately 1:48,000 in the general population, with a male: female ratio of about 4:1 [81]. This condition has a complex genetic background and is linked to abnormalities in the action, differentiation, or migration of GnRH-secreting neurons from the olfactory placode to the hypothalamus during embryonic development; this leads to a reduced production of gonadotropins and, consequently, also of sexual steroids [81]. The role of sex steroids in maintaining bone health is well established, and hypogonadism is widely recognized as a major risk factor for developing osteoporosis [82, 83]. In a 1996 case report, Taylor et al. described a patient with Kallmann syndrome who presented with multiple fractures, with hypogonadism being the only identifiable cause of osteoporosis [84]. Radi et al. later reported a similar case of a 37-year-old man with multiple fractures and a history of pubertal induction therapy, who was later diagnosed with Kallmann syndrome [85]. It has been hypothesized that the absence of sex steroids during pubertal development impairs attainment of normal peak BMD, leading to a reduced bone reserve and increased osteoporosis risk [82, 86]. Several studies have therefore evaluated the effects of androgen replacement therapy on BMD in these patients. The findings suggest that BMD improves with replacement therapy, particularly in patients who adhere consistently to long-term treatment, while those with irregular or insufficient adherence experience less benefit [82, 83, 86, 87]. Maione et al. compared BMD in untreated patients with hypogonadotropic hypogonadism diagnosed after age 40 to age-matched patients diagnosed and treated before age 25. No significant difference in BMD was found between the two groups [88]. However, the possibility could not be excluded that “untreated” patients may have received intermittent hormonal therapy or that the “treated” group may not have been fully compliant with their treatment regimen [88]. Laitinen et al. assessed the relationship between TRT history and bone health measures in 33 subjects with hypogonadotropic hypogonadism, 26 of whom underwent DXA to evaluate lumbar, femoral, and whole-body BMD, body composition, and vertebral morphology [89]. Patients who received inadequate treatment or experienced prolonged interruptions in therapy had lower BMD compared to those on consistent and adequate hormone replacement, although fracture rates did not differ significantly between the groups. Patients with inadequate treatment also showed higher fat mass and a great prevalence of obesity compared to those adequately treated [89]. Ostertag et al. compared 51 treated patients with hypogonadotropic hypogonadism to 40 healthy controls, evaluating BMD using DXA and bone microarchitecture with HR-pQCT [90]. Treated patients had lower BMD at all sites, reduced cortical and trabecular volumetric BMD (vBMD), and decreased cortical thickness at the radius and tibia despite long-term therapy. These findings suggest that treatment may not fully normalize bone health, potentially due to formulation issues or inconsistent adherence [90]. Notably, patients who began therapy while their epiphyses were still open showed increases in both cortical and trabecular density, whereas those who started after epiphyseal closure demonstrated gains only in cortical bone mass [82, 90]. Early recognition and diagnosis of Kallmann syndrome are therefore essential to initiate treatment during adolescence, allowing attainment of adequate peak bone mass and conferring long-term skeletal benefits. Ongoing follow-up with periodic DXA assessments is necessary to monitor BMD, ensure therapeutic adequacy, and guide any necessary adjustments.

### Androgen deprivation therapy in prostate cancer

Androgen deprivation therapy (ADT) is a mainstay of PC management – whether localized, recurrent, or metastatic – as it can be employed at multiple stages of the treatment pathway and often in combination with other therapeutic modalities [91], leading to an established improvement of survival outcomes in men with PC. Nevertheless, ADT has been linked to significant long-term adverse effects, up to an increased non-cancer mortality [92, 93]. Bone is particularly affected in PC, serving both as a frequent site of metastases (osteoblastic and osteolytic lesions) and as a target of ADT, which accelerates bone turnover favoring bone loss [94]. The molecular mechanisms through which ADT alter bone metabolism in PC are not yet fully understood [95, 96]. Although its detrimental impact on BMD is well established, accumulating evidence indicated that bone quality, rather than bone quantity, plays a predominant role in determining bone fragility [96, 97]. In this scenario, the assessment of BMD assessed by DXA seemed to be an insufficient instrument to capture osteoporosis and bone fragility in most men receiving ADT, as described by Sullivan and colleagues [98]. Hence, many tools have been introduced to assess bone quality and improve fracture risk prediction [60]. Among these, the TBS, a texture parameter derived from lumbar spine DXA images that reflects bone microarchitecture, has been shown to decline early during hormone-deprivation therapies, independently of BMD. Beyond its effect on the bone, the abrupt and profound suppression of androgen and estrogen activity induced by ADT [92, 99] leads to profound and unfavorable body composition changes, including reduced lean mass and increased FBM, often resulting in OSO [94]. DXA derived BC assessment in ADT patients has provided insight on the significant contribution given by BC changes on the fragility risk observed in this population [60, 100], suggesting that ADT-induced BC alterations significantly contribute to the worsening of skeletal health in a peculiar way [95, 96]. Indeed, despite current guidelines support the protective role of higher BMI on bone health [101], recent evidence suggest that in men receiving ADT this protective effect is lost, and obesity is accompanied by both compromised bone quality and accelerated bone loss [96]. Indeed, in obese/overweight hypogonadal men, a reduced androgen availability for aromatization might exert an additional deleterious effect on skeletal health, putting these subjects at particular high risk [96]. As described by the Gruppo Onco-Urologico Piemontese (GOUP) in a cohort of 35 non-metastatic PC-patients receiving ADT, a significant decrease in LBM and a consistent increase in FBM can be observed early after 12 months of treatment [102]. Consistently with the previous evidence, Berruti and colleagues found that adjuvant ADT led to both a significant decrease in BMD and LBM, as well as to an increase in FMB in a prospectively enrolled cohort of 53 non-metastatic PC patients. Interestingly, FMB increase during the first year of treatment, but not during the second year, was found to be a significant predictor of higher risk of skeletal-related events [103]. Recently, Mazziotti and colleagues, demonstrated that vertebral fractures in ADT patients were associated with a T-score lower than − 1DS and with a BMI *≥* 25 kg/m^2^ [104]. In addition, data from the BLADE study indicate that even modest reductions in ALMI during ADT are associated with increased bone turnover and impaired bone formation, suggesting that the reduction of lean mass contributes to bone fragility beyond its effects on falls [105]. Taken all together, these findings highlight the importance of BC changes in addressing skeletal fragility in men receiving ADT. Accordingly, fracture risk assessment should extend beyond BMD to include evaluation of bone qualitative parameters and of LBM and FBM, to more accurately identify high-risk patients and to tailor preventive strategies.

### People living with HIV

Antiretroviral therapy (ART) has transformed the natural history of HIV infection, enabling people living with HIV (PLWH) to achieve life expectancies comparable to those of HIV-negative individuals [106]. Consequently, HIV clinical management now extends beyond viral suppression to the prevention and treatment of non-infectious comorbidities associated with ART and aging [107] including cancer [108], cardiovascular diseases [109], and sexual issues [110]. Although these chronic comorbidities are also observed in the general population, they occur earlier in PLWH [106]. ART-related toxicity was once considered a major contributor; however, current evidence underscores the central role of persistent inflammation, immune activation, and immunosenescence in PLWH, even under effective and optimal ART [111–113]. Hypogonadism is a typical age-related condition which is projected to increase among aging PLWH with a prevalence of approximately 20% among men with HIV, though extreme variability in its laboratory and clinical assessment is reported [114]. However, hypogonadism is also frequently observed in younger (< 60 years) PLWH, reflecting multifactorial pathogenesis involving HIV, lifestyle factors, comorbidities, pharmacological effects, and possibly accelerated aging [114]. Specifically, secondary hypogonadism, often associated with hyperprolactinaemia, is consistently more prevalent than primary forms across both pre-ART and ART eras [115–117], Chronic inflammation with excess cytokine activity, direct viral effects, and ART, particularly protease inhibitors that inhibit cytochrome P450–mediated T metabolism, may collectively impair testicular steroidogenesis, gonadotropin secretion, and their peripheral actions [114]. HIV infection, similar to liver disease and aging, is associated with elevated sex-hormone binding globulin (SHBG) levels. This increase can lead to reduced free T despite normal or even elevated total T, thereby masking hypogonadism. Consequently, assessment of free T is warranted in patients with normal or high total T when symptoms suggest androgen deficiency, as biologically active T may still be low [118]. Relying on T alone may miss nearly half of hypogonadism cases, thus, accurate diagnosis and classification of hypogonadism in PLWH require a complete hormonal profile [119]. Similarly, HIV infection and ART are strongly associated with bone loss and increased fracture risk. HIV proteins contribute to HIV-mediated bone loss through upregulation of NF-κB expression, increased tumor necrosis factor-α levels, and enhanced release of inflammatory cytokines, thereby promoting osteoclast maturation [120]. A meta-analysis reported significantly lower lumbar spine and hip BMD, along with increased risks of overall, fragility, vertebral, and wrist fractures in PLWH compared with controls [121]. Consistent with these findings, real-world data confirm a high prevalence of osteopenia/osteoporosis and vertebral fractures (up to 25%) in PLWH despite regular ART and demonstrate that hypogonadism influences vertebral fracture risk more strongly than BMD and independently of it [122]. The most pronounced reductions in BMD have been observed with regimens containing tenofovir disoproxil fumarate (TDF) and protease inhibitors (PIs); therefore, current clinical practice increasingly favors PI- and TDF-sparing regimens [123–125]. Consistent with this evidence, international guidelines recommend routine monitoring of bone health in PLWH [124, 126]. Changes in body composition have been associated to HIV infection and its treatment since the 1980s. HIV and ART can indirectly contribute body composition changes (i.e., lipodystrophy), promoting T-to-E2 conversion and consequent hypothalamic–pituitary dysfunction, leading to secondary hypogonadism [114]. However, as newer ART have fewer adverse effects and the prevalence of lipodystrophy has declined [127], most international guidelines do not mention body composition analysis in the current routinary management of PLWH [124–126]. However, the interplay between gonadal function, body composition, and bone health in PLWH remains incompletely understood [128], yet growing evidence confirms a bidirectional relationship among comorbidities, overall health status, and sex steroids in this population [129–131]. Sarcopenia, defined as reduced muscle mass, continues to be reported among men living with HIV (MLWH) [132, 133]. A related phenotype, sarcopenic obesity, refers to the coexistence of high adiposity with low muscle mass. In the general population, this condition has been associated with cardiometabolic dysfunction and increased mortality [134]. However, diagnostic criteria for sarcopenia have evolved over time, and no global consensus currently exists regarding its definition, or that of sarcopenic obesity, in PLWH [62, 135]. A recent study has applied two operational definitions [128]: ALMI with sarcopenia defined as < 7.26 kg/m², according to Baumgartner’s criteria [136], and ALM/W, with sarcopenia defined as < 28.27%, as suggested by the recent European consensus [62, 137]. While ALMI remains the most widely used index in MLWH [138], the ALM/W definition is gaining traction in the general population [62]. In this study, similar prevalence rates of sarcopenic obesity were detected by ALMI and ALM/W (11.4%, and 12.4% respectively), and MLWH with sarcopenic obesity defined by ALMI were younger (*p* = 0.025) and had longer HIV infection duration (*p* = 0.045) compared with those without sarcopenic obesity [128].

### Assigned male at birth (AMAB) adults

Transgender people are individuals whose gender identity does not align with their sex assigned at birth such that they may require medical treatment to alleviate distress caused by this incongruence [139]. The diagnosis, both in children and adults, is based on specific diagnostic criteria outlined in the Diagnostic and Statistical Manual of Mental Disorders [140]. Traditionally, we can divide people into two categories: AFAB (assigned female at birth) and AMAB. Based on published epidemiological studies, we can estimate a prevalence of transgender persons in the range of 0.3–0.5% with rates that can reach 4.5% if we include gender diverse persons. In several published studied transgender women (AMAB) seem more prevalent than transgender men (AFAB) [141]. Transgender adults receive gender-affirming hormone therapy (GAHT) to improve gender dysphoria and to better align their physical and emotional characteristics with their affirmed gender. It is generally suggested that such persons be managed by a multidisciplinary team capable of monitoring psychological, metabolic and endocrine aspects [142]. The medical approach is not uniform in the different countries and depends on the health care system, availability of estrogens and antiadrogens and relative cost [143]. Among AMAB individuals the typical regimen includes therapy to lower and/or block T (antiandrogens such as cyproterone or spironolactone and GnRH analogs) along with estrogen to induce feminizing effects (e.g., E2 in different formulations). Cyproterone is the most widely used of the anti-androgens in Europe [144]. Both T and E2 play a role in bone homeostasis, but numerous observational studies found that circulating E2, rather than T, levels were better predictors of BMD and fracture in cisgender men [145]. Not only do transgender individual undergoing hormone therapy represent in vivo models that can contribute to the understanding of the mechanisms of bone homeostasis, but bone health remains a key concern in GAHT safety, particularly regarding assessment methods, reference values, and long-term effects of hormonal and surgical treatments [146]. Although there are no data on prevalence of fragility fractures among AMAB individuals before starting GAHT, transgender women have lower bone density, lower muscle mass and strength and lean body mass compared with cisgender men controls. Wiepjes et al. found that among 711 transgender women, 22% had low bone mass for age (Z-score < − 2.0) at baseline, based on the male reference range [147]. While this approach reflects the sex assigned at birth at the time of assessment, it should be noted that, for clinical fracture risk evaluation, female reference ranges are generally recommended. Therefore, the reported prevalence of low bone mass may vary depending on the reference dataset used [147]. This highlights the ongoing uncertainty regarding the most appropriate reference standards for BMD interpretation in transgender individuals. The cause of low bone density in transgender women is poorly understood but some evidence suggests potential risk factors including poor nutrition, vitamin D deficiency, limited physical activity and body composition [148]. Oestrogen hormone therapy in transgender women has been shown to increase BMD at the femoral neck, radius, lumbar spine, and total body, indicating that skeletal status is preserved during gender affirming therapy, despite substantial muscle loss [149]. A meta-analysis and systematic review of 392 transgender women showed that there was a statistically significant increase in lumbar spine bone density at 12 months and 24 months after initiation of GAHT [150]. The Endocrine Society Practice Guidelines recommend that clinicians obtain BMD for screening purposes in transgender women when risk factors for low bone density exist (such as family history of osteoporosis, smoking, low body weight, chronic corticosteroid use and excessive alcohol use) specifically in those who stop sex hormone therapy after gonadectomy [142]. There are limited data from primary research studies evaluating whether clinicians should use assigned sex at birth or affirmed gender for the determination of T-score or Z-score. Current recommendations are primarily based on expert consensus guidelines, in particular the International Society for Clinical Densitometry (ISCD) Official Positions, which recommend assessing BMD in transgender youth using reference datasets for their perceived gender while accounting for the impact of differing hormonal milieus, as seen in postmenopausal women with or without hormone replacement therapy [151]. In addition to hormonal influences, body composition represents a major determinant of bone health in AMAB transgender individuals. To date, only two studies have systematically analyzed the relationship between BMD and the conventional parameters of body composition assessed by DXA, namely ALM and appendicular skeletal muscle mass index (ASMMI). These works, conducted respectively by Fighera et al. 2018 and Ceolin et al. 2024, have provided preliminary but significant evidence on the role of LBM and FBM as determinants of BMD in transgender populations, with differences related to sex assigned at birth [152, 153]. Fighera et al. 2018 reported a positive correlation between lumbar spine BMD and both ALM, a conventional parameter, and FBM, with ALM and FBM identified as independent predictors of lumbar BMD. However, in this study FBM was assessed in kilograms rather than as a percentage, which is not the conventional DXA output. At the femoral site, age and FBM (kg) emerged as the main determinants of total femur BMD [153]. Similarly, Ceolin et al. 2024 investigated the association between body composition and BMD in the COMET study, reporting that in AMAB individuals only the FMI was significantly correlated with both lumbar and femoral BMD, whereas ASMMI did not show significant associations [152]. It should be noted that FMI, like total FBM, is a non-conventional DXA parameter. Taken together, these findings suggest that in AMAB individuals, FBM (whether expressed as total mass or as FMI) plays a predominant compensatory role in bone health, while appendicular muscle mass (ALM/ASMMI) appears to be less relevant. This contrasts with findings in AFAB individuals, where lean mass parameters show stronger associations with BMD. Such evidence underscores the importance of differentiating between conventional and non-conventional DXA outputs when interpreting body composition–BMD relationships and has relevant clinical implications for the evaluation and prevention of osteoporosis risk in AMAB transgender populations undergoing gender-affirming hormone therapy.

## Supplementary Information

Below is the link to the electronic supplementary material.

Supplementary Material 1 (DOCX 21.3 KB)

## Data Availability

No datasets were generated or analysed during the current study.

## Abbreviations

AFAB: Assigned female at birth
AgRP: Agouti–related peptide
ALM: Appendicular lean mass
ALMI: Appendicular lean mass index
ALM/W: Appendicular lean mass/weight
AMAB: Assigned male at birth
ART: Antiretroviral therapy
ASMMI: Appendicular skeletal muscle mass index
BDNF: Brain–derived neurotrophic factor
BMI: Body mass index
BMC: Bone mineral content
BMD: Bone mineral density
BSI: Bone strain index
CHH: Congenital hypogonadotropic hypogonadism
DXA: Dual–energy X–ray absorptiometry
E2: Estradiol
FBM: Fat mass
FMI: Fat mass index
FSH: Follicle–stimulating hormone
GAHT: Gender–affirming hormone therapy
GH: Growth hormone
GH: IGF1 axis–Growth hormone–insulin–like growth factor 1 axis
GnRH: Gonadotropin–releasing hormone
HR-pQCT: High–resolution peripheral quantitative computed tomography
IGF-1: Insulin–like growth factor 1
IL-1: Interleukin 1
IL-6: Interleukin 6
INSL3: Insulin–like factor 3
KS: Klinefelter syndrome
LBM: Lean body mass
LH: Luteinizing hormone
NPY: Neuropeptide Y
OSA: Osteosarcopenic adiposity
OSO: Osteosarcopenic obesity
PC: Prostate cancer
PLWH: People living with HIV
POMC: Pro–opiomelanocortin
SHBG: Sex hormone binding globulin
SMM: Skeletal muscle mass
T: Testosterone
TBS: Trabecular bone score
TNF-α: Tumor necrosis factor alpha
VFA: Vertebral fracture assessment
VAT: Visceral adipose tissue
Z-score: Number of standard deviations from age–, sex–, and ethnicity–matched mean BMD
T-score: Number of standard deviations from young healthy adult mean BMD
