# Supplementary material for: Body composition in male hypogonadism: practical considerations to the use of dual-energy x-ray absorptiometry
Source: Rev Endocr Metab Disord. 2026 May 25;27(4):875–91. doi: 10.1007/s11154-026-10054-5 (PMC13388776; doi:10.1007/s11154-026-10054-5)
Supplement: Supplementary file 1 — Supplementary Material 1 (DOCX 21.3 KB) [file 11154_2026_10054_MOESM1_ESM.docx]

Supplementary Table 1

| SMM (Kg)  skeletal muscle mass | total mass of skeletal muscle tissue in the body (including muscles of the trunk, limbs, and head) |
| --- | --- |
| SMM/H**^2^** or SMI (Kg/m**^2^** )  skeletal muscle index | total mass of skeletal muscle normalized for height squared |
| SMM/W | total mass of skeletal muscle normalized for weight |
| ALM (Kg) or ASM  appendicular lean mass | total lean mass of the limbs (arms and legs) |
| ALM/H**^2^** or ALMI (Kg/m**^2^** )  appendicular lean mass index | total lean mass of the limbs normalized for height squared |
| ALM/BMI | total lean mass of the limbs normalized for body mass index |
| ALM/W | total lean mass of the limbs normalized for weight |
| FM% (%)  fat mass percentage | ratio of fat mass to total body weight |
| FM/H**^2^** or FMI (Kg/m**^2^**)  fat mass index | fat mass normalized for height squared |
| Android/Gynoid ratio | ratio of fat distribution between the android region (abdomen and upper body) and the gynoid region (hips, thighs, and lower body) |
| % Fat Trunk/Leg  Percentage Fat Trunk-to-Leg Ratio | proportion of fat mass in the trunk region relative to the fat mass in the legs |
| Trunk-to-Limb Fat Mass Ratio | ratio of fat mass in the trunk region to fat mass of the limbs |
| VAT (g, cm^2^ or cm^3^)  visceral adipose tissue | fat stored within the abdominal cavity around internal organs, expressed in g, cm**^2^** or cm^3^ |
